# Novel Tools towards Magnetic Guidance of Neurite Growth: (I) Guidance of Magnetic Nanoparticles into Neurite Extensions of Induced Human Neurons and In Vitro Functionalization with RAS Regulating Proteins

**DOI:** 10.3390/jfb10030032

**Published:** 2019-07-16

**Authors:** Hendrik Schöneborn, Fabian Raudzus, Emilie Secret, Nils Otten, Aude Michel, Jérome Fresnais, Christine Ménager, Jean-Michel Siaugue, Holm Zaehres, Irmgard D. Dietzel, Rolf Heumann

**Affiliations:** 1 Department of Biochemistry II–Molecular Neurobiochemistry, Faculty of Chemistry and Biochemistry, Ruhr-Universität Bochum, 44801 Bochum, Germany; 2 Physico-chimie des Électrolytes et Nanosystèmes Interfaciaux, PHENIX, Sorbonne Université, CNRS, F-75005 Paris, France; 3 Department of Biochemistry II–Electrobiochemistry of Neural Cells, Faculty of Chemistry and Biochemistry, Ruhr-Universität Bochum, 44801 Bochum, Germany; 4 Department of Anatomy and Molecular Embryology, Faculty of Medicine, Ruhr-Universität Bochum, 44801 Bochum, Germany; 5 Department of Cell and Developmental Biology, Max Planck Institute for Molecular Biomedicine, 48149 Münster, Germany

**Keywords:** RAS GTPase, GTP exchange factor, SOS, magnetic nanoparticle, Parkinson’s disease, regeneration, dopaminergic neuron, fluorescence correlation spectroscopy, multiangle light scattering, magnetic nanoparticles

## Abstract

Parkinson’s disease (PD) is a neurodegenerative disease associated with loss or dysfunction of dopaminergic neurons located in the substantia nigra (SN), and there is no cure available. An emerging new approach for treatment is to transplant human induced dopaminergic neurons directly into the denervated striatal brain target region. Unfortunately, neurons grafted into the substantia nigra are unable to grow axons into the striatum and thus do not allow recovery of the original connectivity. Towards overcoming this general limitation in guided neuronal regeneration, we develop here magnetic nanoparticles functionalized with proteins involved in the regulation of axonal growth. We show covalent binding of constitutive active human rat sarcoma (RAS) proteins or RAS guanine nucleotide exchange factor catalytic domain of son of sevenless (SOS) by fluorescence correlation spectroscopy and multiangle light scattering as well as the characterization of exchange factor activity. Human dopaminergic neurons were differentiated from neural precursor cells and characterized by electrophysiological and immune histochemical methods. Furthermore, we demonstrate magnetic translocation of cytoplasmic γ-Fe_2_O_3_@SiO_2_ core-shell nanoparticles into the neurite extensions of induced human neurons. Altogether, we developed tools towards remote control of directed neurite growth in human dopaminergic neurons. These results may have relevance for future therapeutic approaches of cell replacement therapy in Parkinson’s disease.

## 1. Introduction

Neurodegenerative disorders are a major public health issue affecting the worldwide aging population. In particular, Parkinson’s disease (PD) caused by loss and dysfunction of dopaminergic neurons in the substantia nigra of the midbrain is not curable by pharmacological treatment. To this date, symptoms of progressing PD can be retarded mainly by antiparkinsonian medication such as levodopa (L-DOPA) or by deep brain stimulation (DBS) [1]. More recently, a major breakthrough was achieved for patient-specific cell replacement therapies based on the findings by Yamanaka et al., who described four transcription factors to reprogram somatic cells to induced pluripotent stem cells (iPSCs) by retroviral transduction [2,3]. However, regeneration of disrupted neuronal circuits by the transplanted neurons is limited in the adult brain and demands directed axonal regrowth of transplanted neurons into the disconnected target region. Therefore, new approaches are required to remote control cell fiber growth of surviving transplanted neurons upon their neuronal differentiation in situ [4].

Several previous studies focused on the role of rat sarcoma (RAS) triphosphatase (GTPase) signaling in neuronal survival and neurite growth. Borasio et al. showed that intracellular delivered RAS protein promoted survival and fiber outgrowth of peripheral sensory neurons in the absence of neurotrophic factors, nerve growth factor (NGF), or brain derived neurotrophic factor (BDNF) [5]. Conversely, intracellular injection of function-blocking antibody or its monovalent Fab fragments prevented neuronal survival and fiber outgrowth, indicating that neurotrophin-induced RAS activity is sufficient to promote survival and extension of neurites [6]. Moreover, RAS and its downstream effector rapidly accelerated fibrosarcoma (RAF)-kinase are mainly involved in NGF-stimulated axon growth and by RAF1/AKT signaling in axon lengthening, branching, and caliber [7,8].

RAS or its regulators, guanine nucleotide exchange factors (GEFs) and GTPase activating proteins (GAPs), are major players for modulating brain functions such as neuronal connectivity, brain plasticity, and homeostasis [9,10]. More specifically, overexpression of constitutively active Harvey RAS^V12^ (H-RAS^V12^) in neurons of transgenic mice (named synRas) protected adult tyrosine hydroxylase (TH)-positive neurons of the substantia nigra from degeneration induced by dopaminergic neuron-specific toxins [11]. In addition, constitutively active H-RAS^V12^ is a key player in phosphoinositide-dependent kinase 1 (PI3K)-protein kinase B (AKT) signaling, which provides neuroprotective effects by increased expression of anti-apoptotic proteins B-cell lymphoma-extra large (BCL-xL) and B-cell lymphoma 2 (BCL-2) [11], thereby counteracting neurotoxic effects of 6-hydroxydopamine (6-OHDA) in dopaminergic neurons [12]. Taken together, RAS and its regulators may serve as therapeutic targets to promote neuronal survival, neurite growth, and axonal and neuronal network regeneration.

Up to now, no approach has been established to achieve the remote controlled reinnervation of grafted mesencephalic dopaminergic (mDA) neurons into the denervated striatum in PD. Previously, it was shown by Etoc et al. that magnetic nanoparticles (MNPs) functionalized with the RAC1 guanine nucleotide exchange factor TIAM promoted in cell protrusion and actin cytoskeleton remodeling [13].

In this study, we bound purified HaloTag™ derivatives of H-RAS^V12^ or the guanine nucleotide exchange factor domain SOS1cat proteins (with or without Clover) onto HaloTag™ Ligand-functionalized magnetic nanoparticles (HTL-MNPs). This was a first step towards our final aim to engineer signaling platforms for fiber growth arising from activation of the inner face of the plasma membrane upon magnetic stimulus. Fusion proteins were characterized by Western Blot, pull-down assay, or GEF activity measurements. The binding of protein onto HTL-MNPs was demonstrated using fluorescence correlation spectroscopy (FCS) and multiangle light scattering (MALS). As we aim to perform all experiments in human dopaminergic neurons, we directly converted human neural progenitor cells (NPCs) to dopaminergic neurons using small molecules [14]. Cells were characterized by immunofluorescence staining and electrophysiological measurements. Moreover, we delivered MNPs into the cytoplasm of induced neurons by microinjection and guided them into neuronal fibers upon an external magnetic stimulus.

## 2. Materials and Methods

### 2.1. Culturing of Human Neural Progenitor Cells

Human NPCs were derived from human fibroblast iPSC by treatment with small molecules as described before [14,15]. Human induced pluripotent stem cell derived neural progenitor cells used in this study were generated at the Max Planck Institute for Molecular Biomedicine (Münster, Germany) [15]. NPCs were cultured on Matrigel-coated 12-well cell-culture plates (Nunc, Rochester, New York, NY, USA) in N2B27 medium supplemented with 3 µM CHIR99021 (Axon Medchem, Groningen, The Netherlands), 0.5 µM Smoothened agonist (SAG) (Cayman Chemical, Ann Arbor, MI, USA), and 150 µM Ascorbic Acid (AA; Merck, Darmstadt, Germany) with a medium change every second day. For plate coating, Matrigel (high concentration, growth factor reduced; Corning, New York, NY, USA) was diluted 1:100 in Knockout Dulbecco modified eagle medium (DMEM, Thermo Fisher Scientific, Waltham, MA, USA) for coating with 500 µL per well for 2 h. Cells were split in a 1:9 to 1:12 ratio every 7 days by single cell digestion with prewarmed accutase (Merck) for 10 min at 37 °C. Cells were diluted 1:10 in DMEM (Merck) prior to centrifugation at 200× *g* for 5 min. After resuspension in fresh NPC medium (N2B27 supplemented with CHIR, SAG, and AA), cells were plated on Matrigel-coated cell-culture dishes.

### 2.2. Generation of mDA Neurons from NPCs

Midbrain dopaminergic neurons were generated from NPCs by changing medium one day after splitting to N2B27 medium supplemented with 100 ng/mL Activin A (Miltenyi Biotech, Bergisch Gladbach, Germany), 1 µM SAG, and 200 µM AA [14]. After 8 days in this neuronal induction medium, maturation of neurons was achieved by cultivation in N2B27 medium with 1 µM SAG for two more days, 10 ng/mL BDNF (Peprotech, Hamburg, Germany), 10 ng/mL GDNF (Peprotech), 1 ng/mL TGF-*β*3 (Peprotech), 200 µM AA, and 500 µM dbcAMP (Merck). One day after starting the maturation process, cells were split 1:3 by accutase treatment. For analysis, cultures were evaluated by immunofluorescence staining and electrophysiology after two weeks of maturation.

### 2.3. Immunocytochemistry

For immunofluorescence staining, cells were plated on Matrigel-coated glass coverslips (Marienfeld Superior, Lauda Königshofen, Germany) in 12-well cell-culture dishes. After removing the medium and rinsing with phosphate buffered saline (PBS), cells were fixed with 4% paraformaldehyde (PFA) in PBS (pH 7.4) for 10 min. Cells were washed three times with ice-cold PBS-T (0.1% Tween 20). Permeabilization was performed by incubation with 0.25% Triton X-100 in PBS for 10 min and for staining membrane proteins by using 0.5% Tween 20 in PBS. Following three washing steps with PBS for 5 min, cells were incubated with 1% bovine serum albumin (BSA) in PBS-T for 30 min to block unspecific binding of the antibodies. Subsequently, primary antibodies in 1% BSA in PBS-T were applied at 4 °C overnight. The next day, coverslips were washed three times in PBS for 5 min. Secondary antibodies in 1% BSA in PBS-T were applied for 1 h at room temperature. Cells were counterstained for nuclei by incubation with PBS containing 1 µg/µL Hoechst 33342 (Thermo Fisher Scientific) for 5 min and then washed thrice with PBS. Finally, coverslips were mounted on microscope slides (Thermo Fisher Scientific) with Aqueous Mounting Medium (Abcam, Cambridge, United Kingdom) and imaged using an Olympus IX83 inverted fluorescence microscope or Leica SP8 confocal microscope. In this study, the following antibodies were used: rabbit anti-TH (0.6 µg/mL; Merck), mouse anti-*β*-Tubulin III (1 µg/mL; STEMCELL Technologies, Vancouver, Canada), mouse anti-Nestin (0.25 µg/mL; STEMCELL Technologies), rabbit anti-PAX6 (4 µg/mL; STEMCELL Technologies), rabbit anti-SOX1 (2 µg/mL; STEMCELL Technologies), rat anti-DAT (~100 ng/mL; Merck), mouse anti-PSD95 (2 µg/mL; Thermo Fisher Scientific), rabbit anti-Synaptophysin (1 µg/mL; Abcam), and CD47-PE (5 µg/mL; Miltenyi Biotech). Secondary antibodies were conjugated to AlexaFluor fluorochromes 488, 555, 594, 633, and 647 (0,5 µg/mL), and all were obtained from Thermo Fisher Scientific.

### 2.4. Patch-Clamp Recordings of Induced Dopaminergic Neurons

Na^+^ and delayed rectifier K_dr_ currents were recorded from NPC-derived neurons after two weeks of differentiation according to the protocol using whole-cell patch-clamp configuration. The patch pipettes were fabricated from borosilicate glass capillaries (GB-150TF-8P, Science Products, Hofheim, Germany) on a PP-830 puller (Narishige Europe, London, UK) with resistances of 6–10 MΩ after filling with pipette solution. For Na^+^ current quantification, we used a pipette solution containing: CaCl_2_ 0.1 mM, CsF 100.0 mM, EGTA 1.1 mM, HEPES 10.0 mM, MgCl_2_ 5.0 mM, and NaCl 5.0 mM. Cells were perfused in a bath solution containing: 4-Aminopyridine 4.0 mM, CaCl_2_ 1.0 mM, CdCl_2_ 0.5 mM, glucose 10.0 mM, HEPES 10.0 mM, MgCl_2_ 1.0 mM, NaCl 100.0 mM, and TEA-Cl 10.0 mM. For K_dr_ measurements, the pipette solution contained: CaCl_2_ 0.1 mM, EGTA 1.1 mM, glucose 25.0 mM, HEPES 10.0 mM, K^+^-gluconate 100.0 mM, Mg^2+^-ATP 3.0 mM, MgCl_2_ 5.0 mM, and NaCl 5.0 mM. The bath solution consisted of: CaCl_2_ 1.8 mM, glucose 16.0 mM, HEPES 10.0 mM, KCl 5.4 mM, MgCl_2_ 0.8 mM, and NaCl 110.0 mM. The recordings were performed at room temperature using an L/M-EPC7 amplifier (List Medical, Darmstadt, Germany). Signals were filtered using the EPC7 10 kHz lowpass filter and obtained data were analyzed with PClamp 10 software (Version 10.1.0.10, Molecular Devices, Sunnyvale, CA, USA). Due to deteriorating cells accompanied by increasing leakage currents, the maximal measurement period for each cell culture dish was limited to 60 min.

Na^+^ currents were recorded with a series of depolarization steps starting from a holding potential of −77 mV after a liquid junction potential correction of −7 mV in increments of 5 mV. Corresponding to the maximum of current–voltage relationship, maximal peak Na^+^ currents were determined at a test potential of −12 mV. Voltage-dependent inactivation of Na^+^ currents were evaluated by a series of prepulses with the duration of 200 ms in 5 mV increments starting at an initial hyperpolarization potential of −82 mV followed by a depolarization step to a test potential of −17 mV for triggering the maximal Na^+^ currents. For steady-state inactivation of Na^+^ currents, peak Na^+^ currents were fitted versus prepulse potential to a modified Boltzmann equation with *I*_0_ as the current elicited from most negative prepulse potential, *V_In_*_1/2_ as the prepulse potential at which half of the channels are inactivated, and slope factor *S*:

(1) $$\frac{I}{I_{0}}=\frac{1}{1+exp\left[\frac{\left(V_{m}-V_{In1/2}\right)}{S}\right]}$$

Using a P/4 protocol, leakage and capacitive artifacts were subtracted. Na^+^ current densities were calculated by normalization of peak Na^+^ current to the cell capacitance. To minimize errors of poor membrane voltage control, only cells with leak currents not surpassing 100 pA and their series resistances 20 MΩ as well as Na^+^ currents with an activation voltage range of at least 20 mV in the *I/V* curve were included into the calculation. The liquid junction potential of −7 mV was corrected manually regarding the bath solution.

K_dr_ currents were recorded using a series of step depolarizations of 60 ms starting at a holding potential of −85 mV in increments of 5 mV up to +45 mV. Only cells with a leak current not surpassing 100 pA were included in the statistical evaluation. The current densities were normalized to the capacitance according to the calculation of the Na^+^-current measurements. For determination of the K_dr_ currents, we used the current between 50 ms and 60 ms at +15 mV. The liquid junction potential of −15 mV was corrected manually. All graphs were done using Origin Pro (Origin Lab Corporation, Northampton, MA, USA).

### 2.5. Protein Expression and Purification of HaloTag™ Fusion Proteins

**Figure 1:**
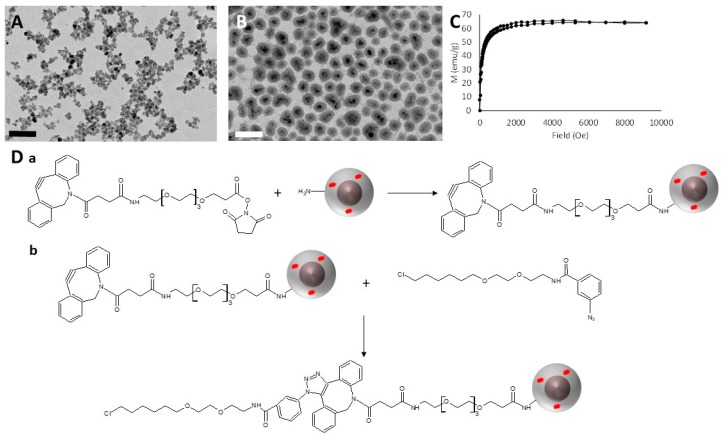
Characterization and functionalization of the magnetic core-shell nanoparticles. (**A**,**B**) TEM images of (**A**) maghemite cores and (**B**) γ-Fe_2_O_3_@SiO_2_ core-shell nanoparticles. Scale bars are 100 nm. (**C**) Magnetization curves obtained by superconducting quantum interference device (SQUID) magnetometry of the maghemite cores. The various curves show data obtained from magnetic fields increasing from 0 to 10,000 Oe and decreasing back to 0. (**D**) Scheme depicting the 2-step HaloTag™ Ligand (HTL) functionalization on the surface of the γ-Fe_2_O_3_@SiO_2_ core-shell nanoparticles by click chemistry. Step (a) corresponds to the dibenzocyclooctyne (DBCO) functionalization of the magnetic nanoparticles, while step (b) is the coupling reaction between the azido-HTL and the DBCO functionalized nanoparticles by click chemistry.

**Figure 2:**
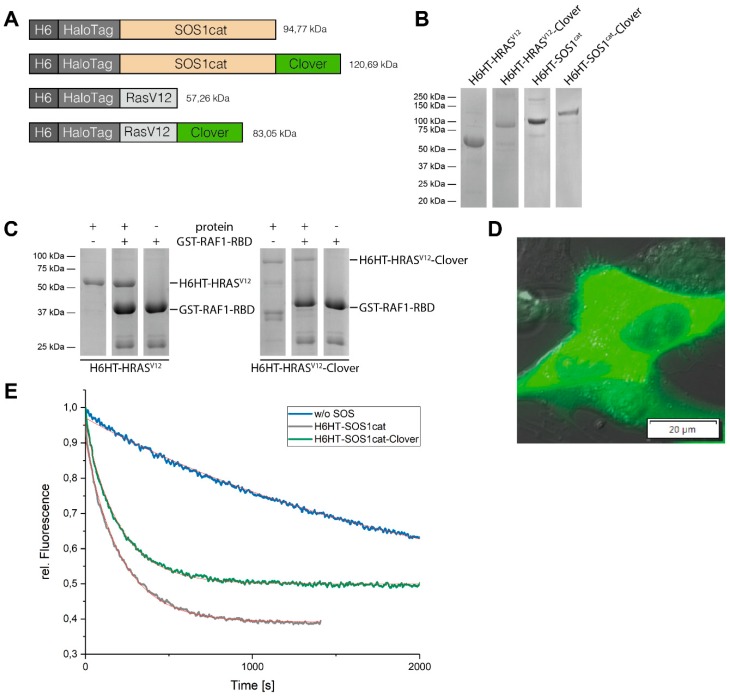
Purification and characterization of HaloTag™-fusion proteins. (**A**) Domain structures of H6HT-H-RAS^V12^ and H6HT-SOS1cat fusion proteins. All fusion proteins have a His6x (H6)-Tag and a HaloTag™ (HT) at the N-terminus in common. In addition, both variants were expressed with the bright monomeric green fluorescent protein Clover. (**B**) SDS-PAGE analysis after purification of HaloTag™-proteins by Ni^2+^-and size exclusion column shows significant bands of each H6HT-fusion protein according to its molecular weight. (**C**) Pull-down of H6HT-H-RAS^V12^ and H6HT-H-RAS^V12^-Clover fusion proteins with RAS-binding domain (RBD) of downstream target rapidly accelerated fibrosarcoma (RAF1). Protein bands of 57 kDa are corresponding to H6HT-H-RAS^V12^ and bands of 83 kDa to H6HT-H-RAS^V12^-Clover. The glutathione S-transferase (GST)-RAF1-RBD band is found around 42 kDa, and its degradation products are at molecular weights around 25–30 kDa. (**D**) Fluorescence image showing Clover fluorescence of H6HT-H-RAS^V12^-Clover fusion protein microinjected into human dopaminergic human derived neuroblastoma cell line (SH-SY5Y) cells. Scale bar is 20 µm. (**E**) GTP exchange factor activity measurements of H6HT-SOS1cat and H6HT-SOS1cat-Clover show the acceleration of mant-GDP/GDP exchange in H-RAS by the order of one magnitude over intrinsic activity in absence of Son of Sevenless 1 (SOS1) cat-fusion protein. Red line indicates “Dissociation-One-phase exponential decay”-fitting curves.

A mutation in bacterial haloalkane dehalogenase (HaloTag™, HT) enables its fusion proteins to covalently bind to the synthetic chloroalkane HaloTag™ Ligand (HTL)-functionalized γ-Fe_2_O_3_@SiO_2_ core-shell nanoparticles (HTL-MNPs, see Figure 1) [16]. These bacterial expression vectors contained at the N-terminus of the fusion protein a His6x-Tag (H6), HT, and either the human sequences of constitutively active H-RAS (H-RAS^V12^) or the catalytic domain of SOS1 (SOS1cat). His6x-Tag facilitated purification and HT enabled covalent binding to the HTL-MNPs. Besides, both constructs were also provided with the bright monomeric green fluorescent protein Clover. Plasmids coding for His6x-HaloTag™ (H6HT)-H-RAS^V12^ (H6HT-H-RAS^V12^), H6HT-H-RAS^V12^-Clover, H6HT-SOS1cat, and H6HT-SOS1cat-Clover were generated by Raudzus et al. (for details, see accompanying manuscript). In short, cDNAs of H-RAS^V12^ and SOS1cat (aa 564-1049) were cloned into the pTriEx™-4 Neo vector (Merck) and fused to the HT-TEV sequence of the pFN18 HaloTag ^®^ T7 Flexi ^®^ vector (Promega, Fitchburg, WI, USA) at the N-terminus using In-Fusion^®^ HD Cloning Plus (Takara Bio Inc., Kusatsu, Shiga, Japan). Additionally, both constructs were established with a sequence coding for bright monomeric green fluorescent protein Clover at the C-terminus. An overview of the constructs is shown in Figure 2A.

After transformation, HT-fusion proteins were expressed in *Escherichia coli* strain BL21 RIL. Then, 100 mL Lysogeny Broth (LB) (Lennox)-medium containing 50 µg/mL ampicillin were inoculated with a colony containing the expression vector and incubated at 30 °C and 120 rpm overnight. After, 50 mL of the overnight culture were added to 2 L Terrific Broth (TB)-medium complemented with 50 µg/mL ampicillin and incubated at 37 °C and 100 rpm until OD_600_ 0.5–0.6 was reached. Subsequently, protein expression was induced with 1.25 mM isopropyl β-D-1-thiogalactopyranoside (IPTG) at 16 °C and 100 rpm overnight. Cells were collected by centrifugation at 4000× *g* and 4 °C for 30 min. The pellet was directly processed for protein purification or blast-frozen in liquid nitrogen and stored at −80 °C.

For protein purification, the cell pellet was resuspended in 7 mL lysis buffer (50 mM Tris-HCl pH 7.9, 5 mM MgCl_2_, 10% Glycerol, 500 mM NaCl) with 25 U/mL Benzonase (Merck) per g wet weight. Cell lysis was done by sonication using a microtip sonicator (Branson, Danbury, CT, USA) alternating between a 60 s burst at 100 W and a 60 s cooling period. By centrifugation at 25,000× *g* for 30 min at 4 °C, cellular debris were removed, and supernatant was collected. Using a fast protein liquid chromatography (FPLC) system (ÄKTA GE Healthcare, Chicago, IL, USA) equipped with a self-packed Ni^2+^-affinity chromatography column based on Ni-NTA agarose beads (Qiagen, Hilden, Germany), H6HT-fusion proteins were purified from the supernatant through their His6x-Tags at a flow rate of 0.5 mL/min. After equilibrating the column with 5 column volumes of lysis buffer, the lysate was applied to the column and washed with lysis buffer until the absorbance A_280_ was stable. Following a washing step with 5 column volumes of washing buffer (50 mM Tris-HCl pH 7.9, 5 mM MgCl_2_, 150 mM NaCl, 5 mM Imidazole, 1 mM DTT), the proteins were eluted with 5 column volumes of elution buffer (see washing buffer, 250 mM imidazole) and collected in 250 µL fractions by a fraction sampler. Of each purification step, a fraction was collected for SDS-PAGE analysis to identify the fractions containing the desired H6HT-fusion protein. For higher purity, the fractions with the desired protein were pooled and applied to a size exclusion column (SEC; Superdex 200 Increase 10–300, GE Healthcare). Proteins were eluted isocratically from the SEC by one column volume of buffer (20 mM HEPES pH 7.4, 50 mM NaCl, 1 mM DTT) at a flow rate of 0.25 mL/min and collected in 200 µL fractions. Finally, fractions containing the H6HT-fusion proteins were identified by SDS-PAGE and Western Blot and pooled prior concentrating with centrifugal concentrators (Sartorius, Göttingen, Germany).

### 2.6. GTP Exchange Factor Activity-Measurement of SOS1cat-Fusion Proteins

The catalytic domain of the GEF Son of Sevenless (SOS) is sufficient to promote GDP/GTP nucleotide exchange in RAS [4]. We purified H6HT-SOS1cat and H6HT-SOS1cat-Clover fusion proteins and measured their GEF activity by observing the fluorescence decrease through the exchange of fluorescent N-Methylanthraniloyl (mant)-GDP in preloaded H-RAS to unlabeled GDP. In a quartz cuvette, mant-GDP-loaded H-RAS was adjusted to a concentration of 1 µM with nucleotide exchange buffer (40 mM HEPES pH 7.4, 5 mM MgCl_2_, 1 mM DTT) for a total reaction volume of 150 µL. The fluorescence intensity was recorded at 440 nm emission wavelength every 2 s by a fluorescence spectrometer. After reaching a stable fluorescent signal, we added 2 µL of a 100 mM GDP solution to measure the intrinsic nucleotide exchange activity. In the following runs, we proceeded in the same way, but we further added 0.5 µM of either H6HT-SOS1cat fusion proteins to the reaction mix, thus determining their impact on nucleotide exchange rates.

For data analysis, we exported the data to an analysis software (OriginPro). We normalized the fluorescence intensity for the first time point after THE addition of GDP to 1 and, according to that, we calculated the relative fluorescence of later time points. Because of the large excess of GDP over H-RAS and the exponential decrease of fluorescence due to mant-GDP release from H-RAS, we calculated a pseudo-first order rate constant (observed rate constant, K_obs_) using a “Dissociation-One-phase exponential decay”-fitting model [17].

### 2.7. Pull-Down of H-RAS^V12^-Fusion Proteins

H-RAS^V12^ binds to the RAS-binding domain (RBD) of its downstream effector RAF1 as a constitutively active GTP-bound variant. After purification (see Section 2.5), 200 µg of H6HT-H-RAS^V12^ and H6HT-H-RAS^V12^-Clover fusion proteins were precipitated with GST-RAF1-RBD fusion protein obtained from RAS Pull-Down and Detection Kit (Thermo Fisher Scientific) and washed three times with 1X Lysis/Binding/Wash buffer. Then, 20 µg of each eluted sample were separated in a 12% acrylamide gel by SDS-PAGE. The protein bands were visualized by Coomassie Blue R-250 staining. The pull-down experiment was carried out according to the manufacturer’s protocol.

### 2.8. Culturing of SH-SY5Y cells and Microinjection with H6HT-H-RAS^V12^-Clover

SH-SY5Y is a human neuroblastoma cell line, which was provided by Fanz-Josef Klinz, University of Cologne. Cells were cultured in medium consisting of a 1:1 mixture of Dulbecco’s modified Eagle’s medium high glucose (DMEM, Sigma-Aldrich, St. Louis, MO, USA) and Roswell Park Memorial Institute medium (RPMI-1640, Sigma-Aldrich), supplemented with 10% fetal bovine serum (FBS, Biochrom GmbH, Berlin, Germany), 1% penicillin/streptomycin (Sigma-Aldrich), and 1% glutamine (Sigma-Aldrich) at 37 °C and 5% CO_2_. After reaching 80% confluence, cells were split 1:10 by single cell digestion with a 1:1:8 mixture of trypsin/ethylenediaminetetraacetic acid (EDTA)/PBS for 10 min. Cells were harvested by centrifugation at 200× *g* for 5 min. The pellet was resuspended in 1 mL fresh complete medium and the number of cells obtained by using a Neubauer chamber. The desired number of cells was seeded in a T75 flask with 10 mL complete medium for culturing or in cell culture dishes for experiments.

To study the Clover fluorescence of H6HT-H-RAS^V12^-Clover, we microinjected the fusion protein (5 µg/µL) into SH-SY5Y cells as described in detail in Section 2.12. We analyzed the Clover fluorescence using an inverted fluorescence microscope Olympus IX83 (Olympus, Shinjuku, Tokio, Japan).

### 2.9. Synthesis and HaloTag™ Ligand Functionalization of γ-Fe_2_O_3_@SiO_2_ Core-Shell Nanoparticles (HTL-MNPs)

#### 2.9.1. Synthesis of γ-Fe_2_O_3_ Cores

The sorted maghemite nanoparticles were obtained by an inverse Massart’s co-precipitation method. An acidic equimolar iron (II) and iron (III) ions solution (124.25 g of FeCl_2_, 50 mL of HCl 37%, 250 mL of DI water, 293.5 mL of FeCl_3_ 27%) was added dropwise over 4 h to 2 L of 5% ammonia in water under agitation. After rinsing with deionized water, the obtained Fe_3_O_4_ nanoparticles were redispersed in 360 mL of nitric acid (52%). Then, these nanoparticles were oxidized into γ-Fe_2_O_3_ nanoparticles by boiling the solution with a solution of iron (III) nitrate (323 g in 800 mL of deionized (DI) water) for 30 min. After washing the resulting nanoparticles with nitric acid once (52%, 360 mL) with acetone 3 times and with diethylether twice, they were finally redispersed in DI water, resulting in γ-Fe_2_O_3_ nanoparticles polydispersed in size. To decrease the polydispersity, 10 mL of nitric acid (52.5%) were added to the solution. The addition of nitric acid increased the ionic strength, leading to the flocculation of the larger, thus less stable, nanoparticles. These precipitated nanoparticles were separated from the rest of the ferrofluid, rinsed with acetone and diethyl ether, and finally redispersed in DI water. To ensure their stability and dispersion at neutral pH, the nanoparticles were finally citrated by heating them with sodium citrate for 30 min. After being rinsed with acetone and diethylether, the resulting nanoparticles were dispersed in DI water. The final iron concentration was found equal to 1.25 mol/L. The obtained maghemite nanoparticles were characterized by transmission electron microscopy (TEM) on a JEOL 1011 instrument and by superconducting quantum interference device (SQUID) magnetometry on a Quantum Design MPMS-XL instrument. 

#### 2.9.2. Synthesis of γ-Fe_2_O_3_@SiO_2_ Core-Shell Nanoparticles [18]

Subsequently, 100 µL of the solution of γ-Fe_2_O_3_ nanoparticles were dispersed in 5 mL of DI water and 10 mL of ethanol. Then, 141.9 µL of tetraethylorthosilicate (TEOS, Merck), 250 µL of a 30% ammonia solution, and 23.7 µL of aminopropyltriethoxysilane (APTS, Sigma-Aldrich) functionalized rhodamine B (Sigma-Aldrich) were added to the solution. After 2 h of agitation, the functionalization of the silica shell by polyethylene glycol (PEG) chains and amine functions was carried out by the addition of 49.7 µL of TEOS, 51.7 µL of 3-[methoxy(polyethyleneoxy) propyl] trimethoxysilane (PEOS, ABCR, Karlsruhe, Germany), and finally 25.1 µL of APTS. The mixture was stirred overnight. The resulting nanoparticles were then rinsed 3 times with a mixture of diethylether/ethanol 15:1 and finally redispersed in 5 mL of a 3-morpholinopropane-1-sulfonic acid (MOPS) buffer at 0.1 mol/L and pH = 7.4. The obtained core-shell nanoparticles were characterized by TEM.

#### 2.9.3. Functionalization of the γ-Fe_2_O_3_@SiO_2_ Core-Shell Nanoparticles with HaloTag™ Ligands [19]

Then, 1 mL of the dispersion of core-shell particles in MOPS was mixed with 73.6 µL of dibenzocyclooctyne-PEG4-N-hydroxysuccinimidyl ester (DBCO-PEG-NHS, Sigma-Aldrich) at 0.01 mol/L in dimethylsulfoxyde (DMSO, Sigma-Aldrich) for 1 h. The DBCO-functionalized nanoparticles were then rinsed on Sephadex G-25 steric exclusion columns (PD10 columns, GE Healthcare) with HEPES 0.2 mol/L at pH = 8. They were then mixed with 9.2 µL of the azido-functionalized HaloTag™ Ligand (HTL) (0.01 mol/L in DMSO) overnight. Then, 368 µL of succinic anhydride (Sigma-Aldrich) at 0.5 mol/L in DMSO were added to the dispersion, and the mixture was agitated for 45 more minutes. The HTL-functionalized nanoparticles were then rinsed on steric exclusion columns with HEPES 0.2 mol/L at pH = 8. The non-functionalized nanoparticles underwent the same succinic anhydride process in order to convert all the amine groups into carboxylic acid groups and to result in negatively charged particles that would not stick to negatively charged intracellular membrane.

### 2.10. Protein-Functionalization of Magnetic Nanoparticles Demonstrated by Fluorescence Correlation Spectroscopy

Fluorescence correlation spectroscopy (FCS) is a powerful technique to measure diffusion rates by correlation analysis of temporal fluctuations of the fluorescence intensity, thereby gaining insights on the protein adsorption layer around MNPs with subnanometer precision. In particular, quantification of intensity changes caused by fluorophores passing a detection volume of about 1 fL in a confocal microscope lead to an average diffusion time [20].

FCS allows measuring diffusion rates by correlation analysis of temporal fluctuations of the fluorescence emission intensity due to variations in concentration of fluorescent molecules in the observation volume. For monomers and small molecules, high-frequency fluctuations are observed, whereas oligomers and particles with increased size show low-frequency fluctuations. These intensity changes are quantified by temporally auto-correlating the recorded intensity signal. The autocorrelation function *G* (*τ*) is fitted versus lag time *τ*. Since we expect three-dimensional (3D) Gaussian approximation, we describe the free diffusion of a single type of particles in three dimensions with a model for *G* (*τ*) [21].

(2) $$G\left(\tau \right)=\frac{1}{N}\frac{1}{1+\left(\frac{\tau }{\tau_{D}}\right)}\sqrt{\frac{1}{1+\left(\frac{\tau }{\tau_{D}}\right){\left(\frac{\omega_{0}}{\omega_{z}}\right)}^{2}}}$$

This model contains $\tau_{D}$ and number of particles *N* as readout parameters of the fit and $\omega_{0}/\omega_{z}$ as the structure parameter determined by calibration. Consequently, size differences of molecules leads to shifts in the autocorrelation function, while the number of molecules in the detection volume influences the amplitude of the autocorrelation function.

The concentration of H6HT-fusion proteins was adjusted to 1 µM, and MNPs with and without HTL were diluted to a final concentration of 5 nM in DPBS. Fluorescein-labeled BSA without HaloTag™ and thereby unable to bind to HTL-MNPs served as negative control. Each fusion protein was mixed in a 1:1 ratio with HTL-functionalized or non-functionalized MNPs as an individual sample and equilibrated for 10 min. Before applying the sample on a 35 mm glass bottom cell-culture dish, the glass bottom was flamed with a Bunsen burner in a laminar flow hood to remove fluorescent impurities. FCS measurements were performed at 25 °C on a Leica TCS SP8 at 594 nm wavelength in combination with the multichannel picosecond event timer and TCSPC module HydraHarp 400 (PicoQuant, Berlin, Germany). Data were analyzed using the SymphoTime 64 software and a 3D Triplet autocorrelation function (PicoQuant).

### 2.11. Protein-Functionalization of Magnetic Nanoparticles Demonstrated by Multiangle Light Scattering

Multiangle light scattering (MALS) is also known as photon correlation spectroscopy (PCS) and quasi-elastic light scattering (QELS), which allows the determination of particle size and its hydrodynamic radius. Macromolecules are buffeted by solvent molecules, leading to Brownian motion. Light is scattered by moving macromolecules. Adding the scattered light of two or more particles results in changing destructive and constructive interference, which leads to time-dependent fluctuations in the intensity. These fluctuations are quantified by a second order correlation function $G^{2}\left(\tau \right)$ with I(t) as the intensity of scattered light at time *t*. Average overall *t* is indicated by pointed brackets:

(3) $$G^{2}\left(\tau \right)=\frac{\langle I\left(t\right)I\left(t+\tau \right)\rangle }{\langle I{(t)\rangle }^{2}}$$

Since we assume a monodisperse solution, the correlation function is described with baseline *B* at infinite delay *β*, as the correlation function amplitude at zero delay and the decay rate Γ as:

(4) $$G^{2}\left(\tau \right)=B+\beta e^{-2\Gamma \tau }$$

With the relation $D=\frac{\Gamma }{q^{2}}$ describing the diffusion constant *D* and the magnitude of the scattering vector *q*, the hydrodynamic radius $r_{h}$ was calculated by the Stokes–Einstein equation, where *k* is Boltzmann’s constant, *T* is the temperature in K, and η is the solvent viscosity:

(5) $$r_{h}=\frac{kT}{6\pi \eta D}$$

MNPs were tested for increase of hydrodynamic radii after protein binding and thus for protein-functionalization. MALS was measured for 1.2 nM HTL-functionalized and non-functionalized MNPs alone and separately loaded with 10 µM Fluorescein-labeled BSA, H6HT-H-RAS^V12^, or H6HT-SOS1cat-Clover fusion protein using a WYATT miniDAWN TREOS II system (Wyatt, Santa Barbara, CA, USA). Fluorescein-labeled BSA served as negative control since it is not able to bind to HTL.

### 2.12. Microinjection and Remote Controlling of Magnetic Nanoparticles in NPCs and Induced DA Neurons

MNPs were microinjected into NPCs and induced DA (iDA) neurons. Therefore, we used microcapillaries (FemtoTips I and II, Eppendorf, Wesseling-Berzdorf, Germany) with an inner diameter of 500 nm in combination with a programmable microinjector with integrated pressure supply (FemtoJet 4i, Eppendorf) and a micromanipulator with dynamic movement control (InjectMan 4, Eppendorf) mounted to an inverted fluorescence microscope. Prior to the MNP injection, MNPs were separated to a monodisperse solution by sonication for 4 min in a sonication bath (Allpax, Papenburg, Germany), and aggregates were removed by centrifugation at 4000× *g* for 1 min. Supernatant containing disperse MNPs were loaded into the microcapillaries using microloader pipette tips (Microloader, Eppendorf) suitable for 10 µL pipettes. The injection of the MNPs was performed for 0.45 s with 25 hPa injection pressure and 20 hPa compensation pressure at an injection angle of 35°. To remote control intracellular MNPs, a magnetic tip was attached to the front of the microloader instead of an injection needle and then smoothly approached to an injected cell with micrometer precision. For a magnetic tip with a high magnetic gradient, an iron piano string of 0.6 mm in diameter was pulled apart in a flame of a Bunsen burner to form a fine tip tapering to a point. The front-end of the pulled tip (3 mm long) was placed at the pole of a Neodymium-Ferrum-Boron (NdFeB) magnet (size 2 mm × 1 mm × 5 mm; 44.785 MGsOe) and together plugged in a modified pipette tip fitting to the micromanipulation system. The intracellular localization and dynamics of the MNPs doped with Rhodamine fluorescence were observed by an inverted fluorescence microscope (Olympus IX83, Olympus, Shinjuku, Tokio, Japan).

## 3. Results

### 3.1. Synthesis and HaloTag™ Ligand Functionalization of γ-Fe_2_O_3_@SiO_2_ Core-Shell Nanoparticles (HTL-MNPs)

The synthesis of the magnetic nanoparticles used in this work was a two-step process. In the first step, maghemite γ-Fe_2_O_3_ nanoparticles were obtained by an inverse co-precipitation of iron salts in an ammonia solution. After size sorting of the synthesized nanoparticles, their average diameter, measured by TEM images analysis (Figure 1A), was 8.8 nm. The saturation magnetization of the maghemite cores was measured by SQUID magnetometry at 64.8 emu/g (Figure 1C). These cores were then covered by a thick silica shell (Figure 1B), in which was also encapsulated a fluorescent molecule, Rhodamine B, to allow their visualization by fluorescence microscopy once inside cells. The surface of those nanoparticles was functionalized by short PEG chains and amine groups in order to ensure their colloidal stability through steric hindrance and electrostatic interactions. The physical size of the γ-Fe_2_O_3_@SiO_2_ core-shell nanoparticles, measured by TEM images analysis, was 49.9 ± 12.7 nm (measurements performed on 215 particles). The amine groups also allowed the further functionalization of the nanoparticles with a HaloTag™ Ligand through click chemistry (Figure 1D).

### 3.2. Purification of HaloTag™-Fusion Proteins and their Characterization

HaloTag™-fusion proteins H6HT-H-RAS^V12^ (57.26 kDa), H6HT-H-RAS^V12^-Clover (83.05 kDa), H6HT-SOS1cat (94.77 kDa), and H6HT-SOS1cat-Clover (120.69 kDa) were expressed in *E. coli* and purified by an Ni^2+^-column and a size exclusion column using fast protein liquid chromatography (Figure 2A). The expression and the purification of the proteins were verified by SDS-PAGE analysis showing significant bands for each protein according to its molecular weight (Figure 2B). The interaction of H6HT-H-RAS^V12^ and H6HT-H-RAS^V12^-Clover proteins with the RAS-binding domain (RBD) of their downstream signaling partner RAF1 was shown by a pull-down assay (Figure 2C). Purified H6HT-H-RAS^V12^ ± Clover fusion proteins were enriched by affinity purification using GST-RBD-fusion proteins along with glutathione agarose resin. The following SDS-PAGE analysis demonstrated the precipitation of H6HT-H-RAS^V12^ and H6HT-H-RAS^V12^-Clover fusion proteins with GST-RAF1-RBD. Protein bands at 57 kDa or 83 kDa indicated the pull-down elute of H6HT-H-RAS^V12^ or H6HT-H-RAS^V12^-Clover. GST-RAF1-RBD of the elutes with and without HT fusion proteins was detected at 42 kDa. Its degradation products were identified around 25–30 kDa. Interestingly, neither the N-terminal His6x-Tag and HaloTag™ nor the C-terminal fluorescent protein Clover blocked the binding to RAF1-RBD. The Clover fluorescence of H6HT-H-RAS^V12^-Clover fusion protein microinjected into human dopaminergic SH-SY5Y cells was shown by fluorescence microscopy (Figure 2D). The fluorescence was distributed homogenously throughout the cytoplasm and the neurites. Please note, the partial exclusion of the fluorescence from the nucleus is in agreement with the previously described cut-off of nuclear pores of around 60 kDa for diffusion controlled transfer [22].

The biological activities of H6HT-SOS1cat and H6HT-SOS1cat-Clover were determined by GTP exchange factor activity measurements. We measured the fluorescence decrease of mant-GDP loaded H-RAS protein by exchange to GDP in the absence and the presence of the GTP exchange factor SOS1cat fusion proteins. For analysis, we plotted the relative fluorescence intensity against the time and calculated a pseudo-first order rate constant (observed rate constant, k_obs_) using a “Dissociation-One-phase exponential decay”-fitting model (Figure 2E). In the absence of SOS1cat fusion proteins, we obtained an observed rate constant k_obs_ = 4.42 × 10^−4^ ± 6.90 × 10^−7^ s^−1^ and half-lifetime *τ* = 2,261.48 ± 3.53 s. In the presence of H6HT-SOS1cat, we determined k_obs_ = 4.75 × 10^−3^ ± 2.10 × 10^−5^ s^−1^ and *τ* = 210.31 ± 0.93 s and for H6HT-SOS1cat-Clover, k_obs_ = 4.92 × 10^−3^ ± 1.88 × 10^−5^ s^−1^ and *τ* = 203.30 ± 0.78 s. From these results, we deduced that our SOS1cat fusion proteins accelerated the mant-GDP/GDP exchange by the order of one magnitude, assuming purification of active H6HT-SOS1cat and H6HT-SOS1cat-Clover fusion proteins. C-terminal Clover domain did not affect GTP exchange activity.

### 3.3. Binding of HaloTag™-Fusion Proteins to HaloTag™ Ligand-Functionalized γ-Fe_2_O_3_@SiO_2_ Core-Shell Nanoparticles (HTL-MNPs)

**Figure 3:**
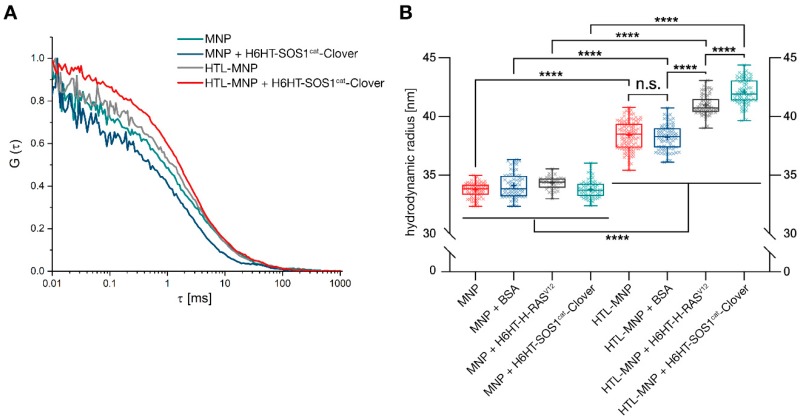
Fluorescence correlation spectroscopy (FCS) and multiangle light scattering (MALS) measurements indicating protein binding of HT fusion proteins to HTL-magnetic nanoparticles (MNPs). (**A**) FCS analysis unraveled the differences in protein-binding of non-functionalized MNPs versus HTL-MNPs. For MNPs, we observed faster diffusion than for HTL-MNPs. Moreover, for HTL-MNPs alone, and especially after incubation with HaloTag™-fusion proteins, we detected decreased diffusion levels. Diffusion levels for MNPs incubated with HaloTag™ fusion proteins remained unchanged compared to MNPs alone. (**B**) For all MNP samples, no changes in the hydrodynamic radii were observed in MALS measurements, assuming that due to the missing HaloTag™ Ligand the incubation with HaloTag™-fusion proteins had no impact on the particle size. However, HTL-MNPs showed a significant increase in the hydrodynamic radius since PEGylation and HaloTag™-functionalization increased particle size. Moreover, treating HTL-MNPs with H6HT-H-RAS^V12^ or H6HT-SOS1cat-Clover proteins led to a further increase in particle size. Hence, we deduced the binding of proteins to HTL-MNPs from the increased hydrodynamic radius due to covalent binding of HT fusion proteins. Statistical analysis was performed with Brown–Forsythe and Welch ANOVA test. **** *p* < 0.0001, * *p* < 0.0332 (n = 3).

We used FCS and MALS to obtain insights into binding of the HaloTag™-H-RAS^V12^-and SOS1cat-fusion proteins to the HTL-MNPs. FCS analysis was performed for HTL-functionalized and non-functionalized MNPs after incubation with the HaloTag™-fusion proteins (Figure 3A). MNPs without HTL functionalization showed faster diffusion compared to HTL-MNPs. For HTL-MNPs incubated with fusion proteins, we observed a decreased diffusion due to protein adsorption on the surface of MNPs. In particular, the autocorrelation function of H6HT-SOS1cat-Clover protein loaded HTL-MNPs was shifted to higher lag times, whereas HTL-MNPs and non-functionalized serving as negative controls remained on higher diffusion levels. Hence, due to the reduced diffusion of protein-functionalized MNPs, we detected the formation of a protein sheath around the HTL-MNPs.

Multiangle light scattering allows the measurement of time-dependent fluctuations in scattered light by a fast photon counter (Figure 3B). We analyzed the hydrodynamic radius $r_{h}$ for MNPs and HTL-MNPs, respectively, and incubated with BSA-Fluorescein, H6HT-H-RAS^V12^, and H6HT-SOS1cat-Clover. The mean hydrodynamic radius obtained from MALS measurements for MNPs (without HTL) was 33.7 ± 0.1 nm. After incubation of MNPs with BSA-Fluorescein (34.1 ± 0.1 nm), H6HT-H-RAS^V12^ (34.3 ± 0.1 nm), or H6HT-SOS1cat-Clover (33.8 ± 0.1 nm), we observed no changes in the hydrodynamic radius due to the absence of HTL. The HTL-MNPs showed an increased radius $r_{h}$ of 38.4 ± 0.1 nm due to the PEGylation and HaloTag™-functionalization. HTL-MNPs mixed with BSA-Fluorescein remained unchanged (38.2 ± 0.1 nm). After incubation of HTL-MNPs with H6HT-H-RAS^V12^ (41.0 ± 0.1 nm) and H6HT-SOS1cat-Clover (42.1 ± 0.1 nm), we observed a significant increase in hydrodynamic radii. Hence, we confirmed HTL-dependent binding of HT-fusion proteins to HTL-MNPs without formation of an additional corona by FCS with MALS measurements.

### 3.4. Differentiation of Neural Progenitor Cells Into Midbrain Dopaminergic Neurons

**Figure 4:**
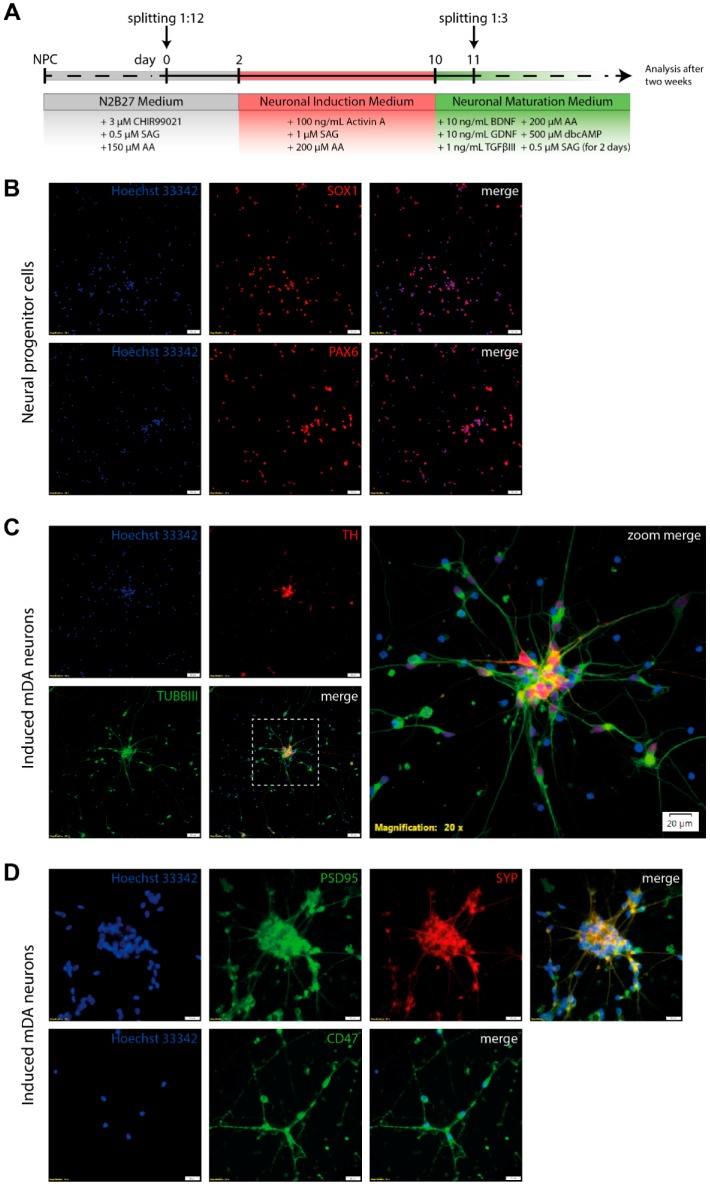
Direct differentiation of human neural progenitor cells into induced mDA neurons. (**A**) Scheme depicting differentiation protocol for this study. (**B**) Immunostaining of neural progenitor cells showed expression of typical markers SOX1 and PAX6. (**C**) Induced mDA neurons were immunostained for TUBBIII and tyrosine hydroxylase (TH), thus demonstrating an expression pattern specifically known for mDA neurons after two weeks of maturation. After differentiation, we obtained 11% TH^+^/Tuj^+^ neurons. (**D**) The induced mDA neurons expressed post-synaptic density protein PSD95, synaptic vesicle glycoprotein Synaptophysin (SYP), and integrin-associated protein Cluster Differentiation 47 (CD47). Nuclei were counterstained with Hoechst 33342. Scale bars are 20 µm. AA = ascorbic acid, SAG = smoothened agonists, BDNF = brain-derived neurotrophic factor, GDNF = glial cell-derived neurotrophic factor, TGF *β*III = transforming growth factor *β*III, dbcAMP = dibutyryl-cyclic AMP.

Human NPCs derived from induced pluripotent stem cells were directly differentiated into mDA neurons by culture with small molecules and cytokines (Figure 4A) [14]. Neural progenitor cells expressed typical markers such as SOX1 and PAX6 demonstrated by immunostaining (Figure 4B). First, we exposed NPCs to Activin A, SAG, and ascorbic acid, thereby leading to a formation of ventral midbrain cells including mDA neurons. After 14 days of maturation, we evaluated the expression of neuronal and dopaminergic marker by immunofluorescence staining. We observed TUBBIII- and tyrosine hydroxylase (TH)-positive neurons, an expression pattern known to specifically mark mDA neurons (Figure 4C). The differentiation efficiency of NPCs into mDA neurons expressing characteristic markers was up to 11%. Interestingly, induced neurons expressed the synaptic vesicle glycoprotein Synaptophysin (SYP), the post-synaptic density protein PSD95, and the integrin-associated protein Cluster Differentiation 47 (CD47) after maturation (Figure 4D).

### 3.5. Electrophysiological Characterization of Induced Neurons

mDA neurons derived from human neural progenitor cells were evaluated for electrophysiological function using whole-cell patch clamping after two weeks of maturation. We distinguished between pyramidal and bipolar neurons and showed peak Na_v_ current densities, average current–voltage relationships normalized to cell capacitances, and the steady-state inactivation of Na_v_ channels fitted with modified Boltzmann equation. In all graphs, for each data point error, bars represent the standard error of mean (SEM).

**Figure 5:**
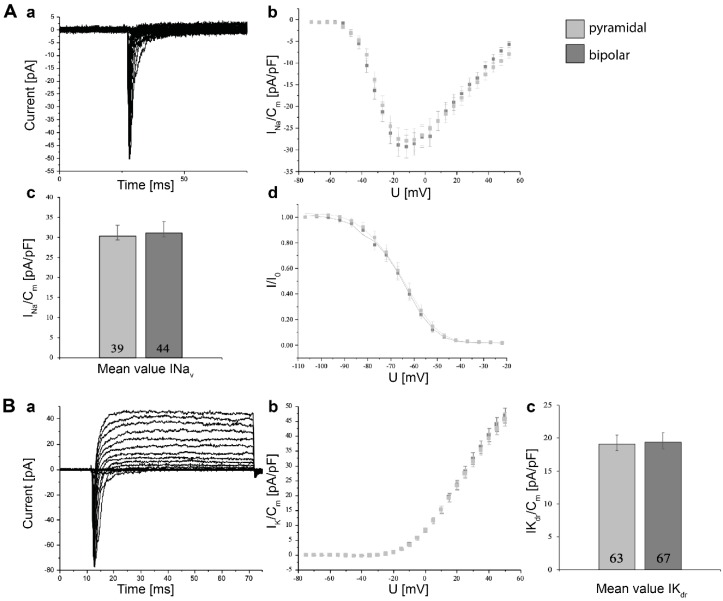
Electrophysiological characterization of neural progenitor cell (NPC)-derived pyramidal-shaped (light-gray) and bipolar (dark-gray) neurons after two weeks of maturation. (**A**) Determination of sodium currents (INav). (**a**) Family of original current recordings. (**b**) Average current–voltage relationships for Na^+^ currents normalized to cell capacitance. (**c**) Peak Na_v_ current densities determined at test potentials of −12 mV starting from holding potentials of −77 mV. (**d**) Steady-state inactivation determined by fitting a modified Boltzmann equation (solid lines) to peak Na^+^ currents obtained after a series of various hyperpolarizing test-pulses. (**B**) Potassium currents (IKdr). (**a**) Family of original current recordings. (**b**) Current–voltage relationships of K_dr_ currents determined by a series of depolarization steps with 60 ms duration starting at a holding potential of −85 mV up to +45 mV. (**c**) K^+^ current densities normalized to cell capacitance. Numbers in bar charts indicate the number of cells recorded from. Error bars represent the standard error of means (SEM).

The peak Na_v_ current densities were averaged and normalized to the capacitances (Figure 5A). The current–voltage relationships of Na_v_ channels followed a bell curve-like shape and allowed a conclusion about the channel opening probability depending on the applied voltage. The maximal current for bipolar neurons was measured at −12 mV being 31.1 ± 2.8 pA/pF (n = 44) and pyramidal-shaped neurons at −12 mV being 30.3 ± 2.7 pA/pF (n = 39). Both steady-state inactivation curves followed a sigmoidal shape inactivating at similar voltages.

For peak K_dr_ current densities, we obtained for bipolar neurons 19.4 ± 1.4 pA/pF in 67 measurements and for pyramidal neurons 19.1 ± 1.4 pA/pF in 63 measurements at a test-pulse potential of +15 mV 50 ms after initiation of the depolarization steps (Figure 5B). The current–voltage relationships of K_dr_ channels showed the outward flow of K^+^ ions due to activation of K_dr_ channels by depolarization of the membrane.

### 3.6. Accumulating Magnetic Nanoparticles in Fibers of Induced Neurons Upon Magnetic Stimulus

**Figure 6:**
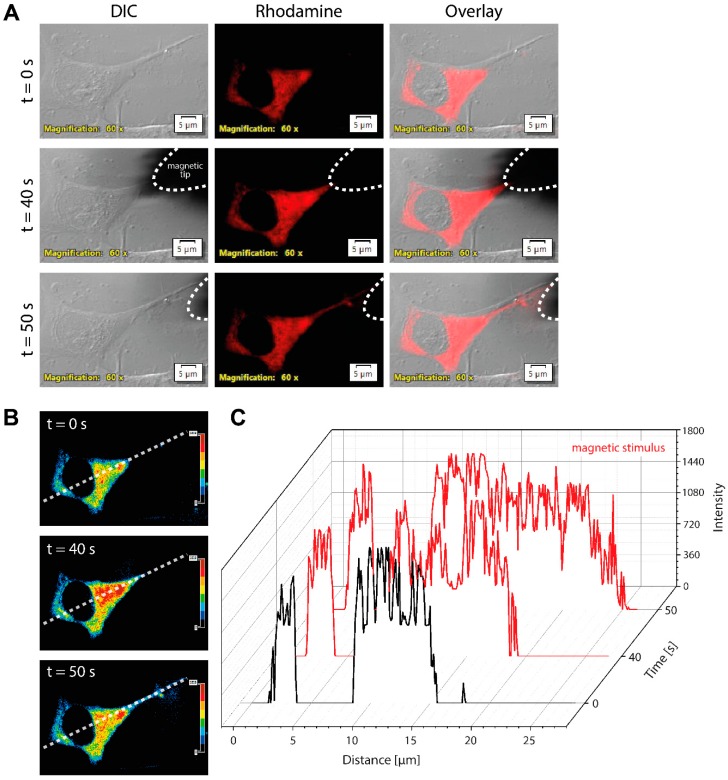
Magnetic nanoparticles accumulated in neuronal fibers of induced mDA neurons upon magnetic stimulus. (**A**) Rhodamine B-doped MNPs were microinjected into induced mDA neurons. By applying a magnetic tip close to the cell (indicated by white dashed line), intracellular MNPs were attracted in the direction of the magnetic field and translocated into the neuronal fiber within 50 s. (**B**) Density maps show the enrichment of MNPs on one site of the cell and in the fiber. (**C**) The intensity plot demonstrates the cytoplasmic distribution of MNPs comparing the three distinct time points along the white dashed line in density maps. By magnetic stimulus (red line), MNPs were guided into the neuronal fiber down to the tip, depicted by the increased distance. Scale bars are 5 µm. DIC = differential interference contrast, *t* = time (s).

Since we aim to control fiber growth by magnetic nanoparticles, we investigated asymmetrical distribution of cytoplasmic MNPs in induced neurons upon magnetic stimulus and their translocation into the neurite tip. Actually, we noted MNPs microinjected into induced neurons accumulating at the inner membrane in accordance with the magnetic field. Rhodamine B fluorescence in the silica shell of MNPs enabled observation using an inverted fluorescence microscope (Figure 6A). Within 50 s of magnetic stimulation, MNPs showed asymmetric localization in the cytoplasm and were observed in the fiber (Video S1). Density maps confirm the enrichment of MNPs on one site of the cell and their translocation down to the tip of a neurite (Figure 6B). An intensity plot of the intracellular MNP distribution compares the MNP intensity along the white dashed line in the density maps at three different time points of magnetic stimulation (Figure 6C). Neurons not stimulated with a magnetic field showed a homogenous distribution of MNPs and no changes in the intensity profile (*t* = 0). The increased intensities over distance after 40 s and 50 s of magnetic stimulation demonstrated the movement of MNPs into the neuronal fibers according to the external magnetic field.

## 4. Discussion

In the present study, we functionalized MNPs with RAS-activity regulating proteins to create signaling tools towards our aim to guide neurite outgrowth by application of remote magnetic forces. We expressed constitutively active H-RAS^V12^ and SOS1cat as fusion proteins with a His6x- (H6) and HT at the N-terminus. For intracellular observation, we additionally purified H6HT-fusion proteins with bright monomeric green fluorescent protein Clover at the C-terminus. H6HT fusion proteins were characterized by biochemical methods and additionally by biological activity (see accompanying paper Raudzus et al.). Fluorescence correlation spectroscopy and multiangle light scattering showed the binding of H6HT-fusion proteins to the surface of MNPs through their HaloTag™ Ligand-functionalization.

In Parkinson’s disease patients, induced DA neurons are currently tested in cell replacement therapies [23,24]. We therefore differentiated human neural progenitor cells into functional mDA neurons and investigated them by immunostainings and electrophysiological measurements. We achieved an asymmetric distribution of MNPs in the cytoplasm of induced neurons by using an external magnetic tip and, moreover, we were able to translocate the MNPs into the neurite.

RAS is involved in neuronal survival [5,25,26], neurite outgrowth [27,28,29], neuronal development of polarity [30], and regeneration [31,32]. During the development of early hippocampal neurons, the longest protrusion between the multiple initial neurites is converted into an axon [33]. Using fluorescence resonance energy transfer (FRET)-based life cell imaging assays, RAS activity was measured during formation of axons. Specifically, H-RAS (but not K-RAS) was spatially confined to the nascent growth cone, and inhibiting H-RAS activity by siRNA resulted in a reduction of polarization. As compared to shorter neurites, H-RAS was preferentially located in the tip of a 200 μm long axon [30]. Moreover, there was a positive feedback loop between H-RAS and phosphatidyl-inositol 3-kinase (PI3K) in the axonal tip. Constructing a PI3K photoswitch, the local control of PIP3 allowed modulation of growth cone dynamics [34]. Altogether, these previous data suggested a functional relevance for enhanced Ras activity found in the tip of the neurites, i.e., axonal growth cone. Hence, RAS and its activator SOS are ideal candidates for manipulating the respective signaling cascades from the inner face of the plasma membrane. We are aware that other small GTPases, cell division cycle 42 (CDC42), RAS-related C3 botulinum toxin substrate (RAC), RAS homolog (RHO), and RAS-like proteins in brain (RAB) GTPases are also involved in neurite outgrowth, guidance, and branching as well as in axon specification [35,36,37,38,39], but RAS activity appears to be upstream of Rho protein signaling, and cross signaling occurs by either cross activation or by overlapping specificities of GTPase-activating proteins and nucleotide exchange factors [4,40]. Here, we focus on H-RAS and SOS, aiming towards oriented fiber growth.

Interestingly, the biochemical analysis of the H6HT-fusion proteins of SOS1cat revealed no inhibitory effects on binding guanine nucleotide affinities or GEF activity either by the N-terminal tags or the C-terminal Clover domain (without SOS: k_obs_ = 4.42 × 10^−4^ ± 6.90 × 10^−7^ [s^−1^]; with H6HT-SOS1cat-Clover: k_obs_ = 4.92 × 10^−3^ ± 1.88 × 10^−5^ [s^−1^]). FCS allowed more detailed insights in protein binding of HT-fusion proteins. Additional corona formation as shown for human serum albumin (HSA) variants onto polymer-coated, fluorescently labeled FePt nanoparticles (10 nm in diameter) was not detected in the protein-functionalized MNPs prepared here [41,42].

Previously, dynamic light scattering (DLS) measurements were performed to analyze proteins, to monitor protein aggregation, and to evaluate protein and polystyrene nanoparticles [43,44,45]. According to our knowledge, there have been no previous studies on protein-functionalized magnetic nanoparticles using DLS or MALS to estimate their size distributions. We demonstrated the increase in hydrodynamic radii for HTL-MNPs after incubation with H6HT-fusion proteins, whereas MNPs remained unchanged in size. The method was even more appropriate to gain insights into chemical modifications of MNPs by PEGylation and HaloTag™-functionalization, which led to an alteration of the solvation shell and therefore of the hydrodynamic radius. Moreover, size differences between H6HT-H-RAS^V12^ (57.26 kDa) and H6HT-SOS1cat-Clover (120.69 kDa) protein-loaded HTL-MNPs were detected in MALS measurements. In accordance with Yallapu et al., we observed no significant changes in hydrodynamic radii for MNPs or HTL-MNPs after incubation with BSA-Fluorescein solution [46]. Satzer et al. combined the analysis of nanoparticle sizes by DLS measurements with the detection of nanoparticle size-dependent conformational changes using circular dichroism spectroscopy [47]. Taken together, we were able to exclude additional protein corona formation by FCS and MALS measurements, underlining safe and effective application of HTL-MNPs used here towards future regeneration therapies.

In order to transmit these nanoactuators to current and future concepts of cell replacement therapies, we differentiated human neural progenitor cells into mDA neurons, achieving an ideal in vitro model system of primary cells. Induced mDA neurons were electrophysiologically characterized by determining peak Na_v_ and K_dr_ current densities using whole-cell patch clamping. Furthermore, the expression of specific markers for a neuronal and, more specifically, a dopaminergic phenotype, i.e., TUBBIII, TH, SYP, and CD47, was shown by immunofluorescence stainings. CD47 is an integrin-associated protein and was previously shown to be a suitable candidate for purification and enrichment of dopaminergic precursor cells [48]. More recently, the dopaminergic phenotype was enhanced by gold and nonviral-based mesoporous nanoparticles, suggesting that there are additional future methods allowing further optimization of the strategy presented here, if necessary [49,50,51]. DA neurons may also be generated directly from fibroblasts by viral transduction of cDNAs coding for transcription factors such as Mash1, Brn2, Myt1l, Lmx1a, and Nurr1 and partially completed with mTOR inhibitor Torin1 or miRNA124 [52,53,54,55]. Although various differentiation protocols were described for generating mDA neurons, we believe that only viral transduction-free and cDNA transfection-free approaches are worthy of consideration for cells in replacement therapies. Besides the small molecules and the cytokines used in this study, cell penetrating peptides of recombinant transcription factors [56,57] or in situ transdifferentiation of astrocytes [58,59,60] avoid negative side effects, consequently being eligible for PD therapies.

Here, we showed the intracellular delivery of iron oxide nanoparticles into induced mDA neurons by microinjection next to the nucleus and nanoparticle translocation into neurites upon applying an external magnetic field. The use of iron oxide nanoparticles in biomedical applications is critically discussed, but the new generations of nanoparticles are already widely used in disease diagnosis and treatment, delivery of vaccines, and as contrast agents in dual and triple modal imaging [61]. Although it is known that various types of nanoparticle induced-toxicity leads to apoptosis, necrosis, or autophagy [62], we observed no harmful effects in secondary cell lines such as SH-SY5Y and PC12 (see accompanying publication Raudzus et al.), HEK293, or HeLa cells (data not shown) or in induced mDA neurons under our conditions. Moreover, we expect the PEGylation to increase biocompatibility and avoid clustering of MNPs [63]. We were able to induce an asymmetrical distribution of the MNPs through their attraction by an external magnetic tip and, even more, we were able to translocate the MNPs along a neurite in induced dopaminergic neurons. As shown by Etoc et al., nanoparticles are not limited in cytoplasmic mobility up to a size of 50 nm [64]. In our MALS measurements, we demonstrated cytoplasmic mobility of protein-functionalized HTL-MNPs with an apparent maximal size of ~42 nm in radius.

Recently, in PD, the relevance of progressive accumulation of iron within the SN has been discussed [65,66]. Here, we use nanoparticles localized in the cytoplasm of stem cell-derived dopaminergic precursor neurons to be transplanted into the human brain. Assuming the transplantation of 5 × 10^6^ cells into the brain, the overall load of iron to the brain will be very limited. In addition, the MNPs used here are coated with silicone, thereby reducing the iron released from within the cell. Nevertheless, the long-term stability of the MNPs in the cytoplasm and in the neurite extensions of dopaminergic neurons still has to be critically evaluated.

Finally, all regeneration therapies such as optogenetics, magnetogenetics, or magneto protein therapy have in common that the activation of signaling pathways enhancing the fiber growth have to be triggered in tissues or organs over long distances in millimeter/centimeter range from outside the patients [4,67,68]. Magnetic nanoparticles require high magnetic gradients to respond, which is thought to be overcome by magnetizable implants [67]. Although optogenetics allows focal activation of fiber growth and has been shown to stimulate the RAS/RAF/MAPK pathway signaling, the penetration of light into the brain is limited [4,69,70,71]. Chen et al. combined both emerging technologies and generated upconverting nanoparticles. These nanoparticles absorb tissue-penetrating near-infrared light and emit wavelength-specific visible light, thereby stimulating dopamine release of genetically tagged neurons, even in deep brain regions [72]. However, to our knowledge, light-induced redirection of neurite growth has not been achieved in the brain yet.

Taken together, in this study, we generated cultured human stem cell-derived dopaminergic neurons and injected synthesized MNPs into their cytoplasm. Furthermore, we biofunctionalized MNPs with H-RAS^V12^ or SOS1cat fusion proteins. In the next experiments, we will investigate and characterize if magnetic translocation of HT-H-RAS^V12^ and HT-SOS1cat loaded MNPs to spatially restricted regions of the cell membrane will be sufficient to induce or modulate neurite outgrowth. These MNPs functionalized with RAS-activity regulating proteins were designed to provide a new concept for improvements in PD treatment and more generally in other brain diseases by grafting MNP loaded neurons into the degenerating brain areas, aiming to guide the regenerating axonal tips into distal target regions using an external magnetic field.

## Supplementary Materials

The following are available online at https://www.mdpi.com/2079-4983/10/3/32/s1, Video S1: Magnetic translocation of MNPs into neurites of induced mDA neurons.

## Abbreviations

6-OHDA	6-Hydroxydopamine
AA	Ascorbic acid
AKT	Protein kinase B
BCL-2	B-cell lymphoma 2
BCL-xL	B-cell lymphoma-extra large
BDNF	Brain-derived neurotrophic factor
Brn2	POU domain, class 3, transcription factor 2
CD47	Cluster of Differentiation 47
CDC42	Cell division cycle 42
cDNA	Complementary DNA
DA	Dopaminergic
dbcAMP	Dibutyryl cyclic-AMP
DBS	Deep brain stimulation
DLS	Dynamic light scattering
DMEM	Dulbecco modified eagle medium
ERK	Extracellular-signal-regulated kinase
FCS	Fluorescence correlation spectroscopy
FPLC	Fast protein liquid chromatography
GDNF	Glial cell line-derived neurotrophic factor
GDP	Guanosine diphosphate
GEF	Guanine nucleotide exchange factor
GST	Glutathione S-transferase
GTP	Guanosine triphosphate
GTPase	Guanosine triphosphatase
H6	His6x-Tag
H6HT	H6 = His6x-Tag, HT = HaloTag™
HEK293	Human embryonic kidney 293 cell line
HeLa	Cervical cancer cell line derived from patient Henrietta Lacks
H-RAS	Harvey rat sarcoma
HT	HaloTag™ derived from bacterial haloalkane dehalogenase
HTL	HaloTag™ Ligand
HTL-MNP	HaloTag™ Ligand-functionalised MNP
iPSCs	Induced pluripotent stem cells
L-DOPA	Levodopa
Lmx1a	LIM homeobox transcription factor 1-alpha
MALS	Multiangle light scattering
MANT	N-Methylanthraniloyl
MAPK	Mitogen activated protein kinase
Mash1	Achaete-scute homolog 1
mDA	Midbrain dopaminergic
miRNA	Micro RNA
MNP	Magnetic nanoparticle
Myt1l	Myelin transcription factor 1-like protein
NdFeB	Neodymium-Ferrum-Boron
NGF	Nerve growth factor
NPC	Neural progenitor cell
Nurr1	Nuclear receptor-related 1 protein
PAX6	Paired box protein Pax-6
PD	Parkinson’s disease
PEG	Polyethylene glycol
PI3K	Phosphoinositide-dependent kinase 1
PSD95	Post synaptic density 95 protein
RAB	RAS-like proteins in brain
RAC1	RAS-related C3 botulinum toxin substrate
RAF1	Rapidly accelerated fibrosarcoma
RAS	Rat sarcoma
RBD	RAS-binding domain
RHO	RAS homolog
SAG	Smoothened agonist
SEC	Size exclusion chromatography
SH-SY5Y	Human derived neuroblastoma cell line
SN	Substantia nigra
SOS1	Son of Sevenless 1
SOX1	SRY-box containing gene 1
SYP	Synaptophysin
TGF-*β*3	Transforming growth factor, *β* 3
TH	Tyrosine hydroxylase
TIAM	T-lymphoma invasion and metastasis-inducing protein
TRKA	Tropomyosin receptor kinase A
TRKB	Tropomyosin receptor kinase B
TUBBIII	Tubulin *β*-3 chain*β*
